# The Role of Sirt1 in Ischemic Stroke: Pathogenesis and Therapeutic Strategies

**DOI:** 10.3389/fnins.2018.00833

**Published:** 2018-11-21

**Authors:** Jun-Fang Zhang, Yu-Lei Zhang, Yun-Cheng Wu

**Affiliations:** Department of Neurology, Shanghai General Hospital, Shanghai Jiao Tong University School of Medicine, Shanghai, China

**Keywords:** Sirt1, deacetylase, ischemic stroke, neuroprotection, sirtuin

## Abstract

Silent mating type information regulation 2 homolog 1 (Sirt1), a nicotine adenine dinucleotide (NAD^+^)-dependent enzyme, is well-known in playing a part in longevity. Ischemic stroke is a major neurological disorder and is a leading cause of death and adult disability worldwide. Recently, many studies have focused on the role of Sirt1 in ischemic stroke. Numerous studies consider Sirt1 as a protective factor and investigate the signaling pathways involved in the process under ischemic stress. However, the answer to whether upregulation of Sirt1 improves the outcome of stroke is still a controversy. In this review, we discuss the role and mechanisms of Sirt1 in the setting of ischemic stroke.

## Introduction

Stroke is the second leading cause of death and a major cause of adult disability worldwide (Cushman et al., 2008; Donnan et al., 2008). Ischemic stroke is the most common type of stroke and accounts for 87% of all stroke cases (Macrez et al., 2011). Vascular recanalization therapies, including tissue plasminogen activator thrombolysis and thromboembolectomy, are currently considered the best therapies but are limited due to a narrow treatment window and several safety concerns (Del Zoppo et al., 2009; Albers et al., 2018; Nogueira et al., 2018; Thomalla et al., 2018). There is no effective treatment for ischemic stroke so far. It is crucial to find new therapies for this major medical problem.

There are two major injuries to the brain. Firstly, a blocked cerebral artery and secondly, the subsequent reperfusion may cause the secondary injury. Penumbra, defined as a zone of tissue surrounding the core of the infarction area, is an important target for researchers and clinicians to find effective therapies (Wang et al., 2012). The occlusion of a cerebral artery can cause the deprivation of oxygen and energy, thereby leading to the dysfunction of cerebral tissue and neuron death. After the early phase of necrosis, the following pathophysiological reactions such as formation of free radicals, changes in gene expression, apoptosis, and inflammation contribute to the delayed phase of tissue damage (Moskowitz et al., 2010; Petegnief and Planas, 2013). However, the ineffectiveness of current therapies indicates that there should be other important mechanisms leading to the pathophysiology of ischemic stroke.

**FIGURE 1:**
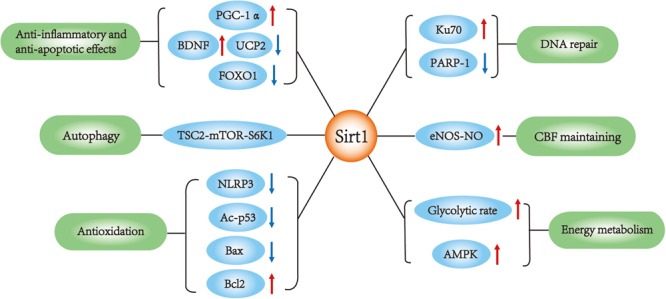
Sirt1 main functions in ischemic stroke. SIRT1 interacts with multiple targets in ischemic stroke, which is involved in the regulation of apoptosis, autophagy, DNA repair, inflammation, metabolism and oxidative stress, cerebral blood flow.

Silent mating type information regulation 2 homolog 1 (Sirt1), also called sirtuin 1, is a nicotine adenine dinucleotide (NAD^+^)-dependent enzyme (Kelly, 2010). Among several potential therapeutic targets, Sirt1 is one of the most valuable candidates because it can modulate gene expression and adapt cell metabolism to ischemic stress. Sirt1 deacetylates numerous transcription factors other than histones and is involved in various biological processes (Chen et al., 2005). A large number of studies consider Sirt1 as a survival factor against aging process, including cardiovascular disease (Li et al., 2011) and neurodegeneration (Donmez, 2012). In recent years, Sirt1 has been found to be neuroprotective against cerebral ischemia/reperfusion (I/R) injury (Herskovits and Guarente, 2014). Activation of Sirt1 alleviates ischemia through several mechanisms (Table 1). Although it is still a controversy whether Sirt1 could improve stroke outcome, there have been plenty of studies indicating the potential therapeutic value of Sirt1 for ischemic stroke. In this review, we discuss the role and potential mechanisms by which Sirt1 protects against ischemic stroke (Figure 1).

**Table 1:** Role and mechanisms of Sirt1 in ischemic stroke.

Compound	SIRT1 role	Mechanism	Reference
IRF9	Anti-apoptosis	IRF9 inhibits Sirt1 deacetylase activity, culminating in the acetylation and activation of p53-mediated cell death signaling in response to acute I/R stress.	Zhang et al., 2017
LKE	Anti-apoptosis, anti-inflammation	LKE mediated, at least in part, through CRMP2 and Sirt1 upregulation and PARP1 inhibition.	Nada et al., 2012
Resveratrol	Anti-oxidation, anti-apoptosis, anti-inflammation	Resveratrol upregulates the Sirt1/PGC-1a, Akt/pCREB, and p38 pathways and downregulates pERK1/2 expression in ischemic injury.	Zhu et al., 2010
	Regulation in glycolytic function	Resveratrol via neuronal Sirt1 promotes glycolytic efficiency to combat energetic stress.	Yue et al., 2008
	Energy regulation	Resveratrol provides neuroprotection by inhibiting PDEs and regulating the cAMP/AMPK/Sirt1 pathway.	Kundu and Thompson, 2008
Resveratrol preconditioning	Anti-oxidation and regulation of neural survival	The mechanism is mediated by Sirt1 through upregulation of BDNF and downregulation of uncoupling protein 2.	Koronowski et al., 2015
HBO-PC	Anti-oxidation	HBO-PC is mediated by the activation of Sirt1 and Nrf2/antioxidant defense pathway.	Xue et al., 2016
Arctigenin	Anti-inflammation	Arctigenin inhibited NLRP3 inflammasome activation through Sirt1 pathway.	Hernandez-Jimenez et al., 2013
Curcumin	Anti-apoptosis	Curcumin activates Sirt1 signaling, resulting in decreased expression of Ac-p53 and Bax and increased Bcl-2 expression.	Miao et al., 2016
Icariin	Anti-oxidation	Icariin protects against brain ischemic injury by increasing the Sirt1 and PGC-1a expression.	Kou et al., 2017
Nampt	Regulation in autophagy	Nampt induces autophagy via TSC2-mTOR-S6K1 signaling pathway in a Sirt1-dependent manner.	Wang et al., 2012
	Energy regulation	Nampt protects against ischemic stroke through rescuing neurons from death via the SIRT1-dependent AMPK pathway.	Dasgupta and Milbrandt, 2007
Leptin	Anti-apoptosis	Leptin increases CB2, Sirt1 and TRPV1 expression as well as expression of the endogenous leptin receptors and reduces the expression of CB1 receptors.	Ashrafi et al., 2017
Magnolol	Anti-apoptosis and anti-inflammation	Magnolol activation of Sirt1 was accompanied by the inhibition of Ac-FOXO1 expression, which decreased the expression of bax and increased Bcl-2 expression.	Chen et al., 2014
Melatonin	Anti-apoptosis	Melatonin increased Sirt1 and reduced Ac-p53 and Ac-NF- κB and was also associated with a rise of Bcl2 and a lowering of Bax.	Wang et al., 2008
Tetrahydroxystilbene glucoside	Anti-oxidation and Anti-apoptosis	The mechanisms are involved with depression of the JNK and Bcl-2 family-related apoptotic signaling pathway, and inhibition of iNOS mRNA expression, which was partly mediated by the activation of Sirt1 and thereby inhibition of NF-κB activation.	Taxin et al., 2014
SalB	Anti-oxidation, anti-apoptosis, anti-inflammation	SalB decreased TNF-α and IL-1 levels in the brain tissue and upregulated the expression of Sirt1 and Bcl-2 and downregulated the expression of Ac-FOXO1 and Bax.	Guo et al., 2017
Estrogen	Energy regulation	Estrogen protects against ischemic stroke via the SIRT1-dependent AMPK pathway.	Wang et al., 2011
HBO-PC	Anti-apoptosis	SirT1 increased Bcl-2 expression and decrease cleaved caspase 3.	Yan et al., 2013
/	Effect on BBB permeability	Sirt1 inhibited Sirt3 expression through the AMPK-PGC1 pathway, causing mitochondrial ROS generation and then increase BBB permeability.	Hurtado et al., 2013
/	Regulation of cerebral blood flow	Sirt1 upregulates the nitric oxide (eNOS–NO) system.	Briski et al., 2014

*IRF9, interferon regulatory factor 9; I/R, ischemia/reperfusion; LKE, lanthionine ketimine 5-ethyl ester; CRMP, collapsin response mediator proteins; PARP, poly-ADP-ribose polymerase; PGC-1α, peroxisome proliferator-activated receptor-gamma coactivator; CREB, cyclic AMP response element-binding protein; ERK, extracellular signal-regulated kinase; NLRP, NOD-like receptor family pyrin domains; Bcl-2, B-cell lymphoma 2; TSC2, tuberous sclerosis complex-2; mTOR, mammalian target of rapamycin; CB, cannabinoid receptor type; TRPV, transient receptor potential vanilloid; FOXO, forkhead box protein O; NF-κB, nuclear factor kappa B; AMPK, adenosine monophosphate (AMP)-activated kinase; JNK, c-Jun N-terminal kinase; BDNF, brain-derived neurotrophic factor; eNOS, endothelial nitric oxide synthase; NO, nitric oxide; iNOS, inducible NO synthase; BBB, blood–brain barrier; HBO-PC, hyperbaric oxygen preconditioning.*

## Anti-Inflammatory and Anti-Apoptotic Effects

Sirt1 plays an important role in endogenous neuroprotection, which is demonstrated using pharmacological and genetic gain of function and loss of function experiments. Activating or inhibiting enzymatic activity of Sirt1 causes a decrease or an increase in infarct volume. Sirt1 is considered to be related to anti-inflammatory and anti-apoptotic effects in cerebral ischemia because its inhibition exacerbated ischemic injury accompanied by increased acetylation of p53 and NF-κB (nuclear factor-kappa B p65), which are important factors mediating inflammatory and apoptotic pathways causing brain damage (Hernandez-Jimenez et al., 2013).

Activation of Sirt1 signaling during cerebral ischemia by curcumin (CCM), a compound mainly extracted from *Curcuma longa*, leads to the decreased expression of Ac-p53 and Bax, increased expression of B-cell lymphoma 2 (Bcl-2), and finally attenuated the inflammation (Miao et al., 2016). Similarly, interventions targeting IRF9 inhibition may help to mediate Sirt1-related ischemic neuron survival through decreased expression of p53 (Chen et al., 2014).

Arctigenin (ARC), a phenylpropanoid dibenzylbutyrolactone lignan derived from *Arctium lappa* L., was reported to provide neuroprotection against ischemic stroke by inhibiting NLRP3 inflammasome activation through the activation of Sirt1 signaling pathway in the middle cerebral artery occlusion (MCAO) model with decreased infarct volume, neurological scores, and brain water content (Zhang et al., 2017). Another study also used MCAO model to demonstrate that LKE (lanthionine ketimine-5-ethyl ester) protected ischemic brain tissue partly through CRMP2 and Sirt1 upregulation and PARP1 inhibition (Nada et al., 2012).

Hyperbaric oxygen (HBO) therapy is considered to be one of the safe and feasible methods to provide neuroprotective benefits for patients with ischemic stroke. It was demonstrated that Sirt1 was involved in the HBO therapy induced ischemic tolerance and Nrf2 might be the downstream regulator of Sirt1 (Xue et al., 2016). Hyperbaric oxygen-preconditioning was found to upregulate the expression of Sirt1 protein and mRNA after focal cerebral ischemic injury, leading to the suppression of apoptosis. Upregulation of Sirt1 caused an increased expression of anti-apopotic Bcl-2 and a decreased pro-apoptotic cleaved caspase-3 in oxygen–glucose deprivation (OGD) injury models (Yan et al., 2013).

## Antioxidation

Cerebral ischemia was found to produce a large number of free radicals and cause neurotoxicity in I/R injury (Crack and Taylor, 2005). Increased generation of intracellular reactive oxygen species (ROS) such as the hydroxyl radical is demonstrated to induce oxidative stress and mitochondrial enzyme dysfunction, leading to the pathophysiology of damage of cerebral ischemia, and this damage, in turn, aggravates cerebral injury (Mattiasson et al., 2003; Slemmer et al., 2008). Therefore, to prevent intracellular calcium accumulation and further cell apoptosis followed by oxidative stress in ischemic stroke, it is critical to find targets to inhibit such cellular signal transduction pathways (Chan, 2004; Mehta et al., 2007; Doyle et al., 2008; Thompson et al., 2015).

Several studies suggested that Sirt1 plays an important role in oxidative stress in ischemic stroke. Peroxisome proliferator-activated receptor gamma coactivator 1-alpha (PGC-1α) is a potent stimulator of mitochondrial respiration and gene transcription in the liver, heart, skeletal muscle, and neurons (Wareski et al., 2009). Hypoxia increases mRNA levels and the protein expression of PGC-1α in wild type mice (Gutsaeva et al., 2008). Increased expression of PGC-1α could reduce neuronal death mediated by oxidative stress (St-Pierre et al., 2006). Sirt1 could directly affect PGC-1α activity through phosphorylation and deacetylation (Canto and Auwerx, 2009), thereby protecting against ischemia stroke.

Resveratrol (3,5,4′-trihydroxystilbene), a polyphenol found in red wine, can ameliorate neuronal damages caused by cerebral ischemia and neurodegenerative diseases such as Alzheimer’s and Parkinson’s disease (Sun et al., 2010; Wang et al., 2014; Pasinetti et al., 2015). Resveratrol was demonstrated to protect ischemic stroke by upregulating the Sirt1-PGC-1α signaling pathways and exert an antioxidative effect under ischemic stress (Shin et al., 2012). Furthermore, resveratrol preconditioning was showed to upregulate BDNF and downregulate uncoupling protein 2 by mediating Sirt1, tolerance in brain (Koronowski et al., 2015). In addition, alpha-lipoic acid (ALA, 1,2-dithiolane-3-pentanoic acid), a free radical scavenger in its oxidized state functions as an essential co-factor in the mitochondrial dehydrogenase complexes. Icariin (ICA), one of the major active flavonoids extracted from the Chinese medicinal herb, *Epimedium brevicornum* Maxim, were also proved to protect against ischemic stroke by increasing Sirt1 and PGC-1α expression (Zhu et al., 2010; Fu et al., 2014).

Another study found that Sirt1 was upregulated chronically at 14 days after a single resveratrol preconditioning treatment. This phenomenon was associated with negative regulation of mitochondrial uncoupling protein-2 (UCP2), a proton channel found in the inner mitochondrial membrane that uncouples oxidative phosphorylation (Della-Morte et al., 2009), by binding directly to its promoter (Bordone et al., 2006) and upregulation of brain-derived neurotrophic factor (BDNF), which is an important growth factor that promotes the survival and growth of neurons (Bramham and Messaoudi, 2005).

In addition, salvianolic acid B (Sal B) (Lv et al., 2015), which is the most abundant and bioactive compound of danshen (*Salvia miltiorrhiza*), and Magnolol (Kou et al., 2017), an organic compound found in the bark of Houpu magnolia (*Magnolia officinalis*), were demonstrated to activate Sirt1 signaling, accompanied by reduced expression of ac- FoxO1 whose function is to synthesize antioxidants and further help neurons provide resistance against oxidative stress (Brunet et al., 2004).

## DNA Repair

As a result of ischemic stroke, oxidative stress will further cause damage to the DNA including oxidative base modifications and strand breaks. The accumulation of oxidative DNA lesions is attributed to the death of neurons. Therefore, the capacity for DNA repair plays an important role in the destiny of neurons in the condition of ischemic stress. Base excision repair (BER) is one of the most crucial parts in the DNA repair systems, which is the endogenous defense mechanism to rescue oxidative DNA damage (Srivastava et al., 1998).

Ku70 is one of the multifunctional DNA repair proteins, which triggers a DNA repair pathway by binding to broken DNA ends including double-strand breaks (Kim et al., 2001). With the decrease of Ku70 after focal cerebral ischemic injury, the repair process was found to be hindered (Kim et al., 2001). Sirt1 is found to regulate the acetylation of Ku70, thereby regulating the DNA repair pathways (Jeong et al., 2007).

Sirt1 also increases the activity of several other DNA repair pathways. The activation of poly [ADP-ribose] polymerase-1 (PARP-1), a key mediator of cell death in excitotoxicity, ischemia, and oxidative stress, contributes to the depletion of NAD^+^ and the release of apoptosis-inducing factor (AIF) from mitochondria, leading to cell death (Alano et al., 2010). The replenishment of cellular NAD^+^ was demonstrated to confer marked neuroprotection effects by enhancing the DNA repair process against ischemic cell death (Wang et al., 2008). Kolthur-Seetharam et al. (2006) reported that SIRT1 modulates PARP-1 activity upon DNA damage. Sirt1 upregulation by resveratrol can reduce PARP-1 activity, however, there is a drastic increase in PAR synthesis leading to AIF-mediated cell death in the sirt1-null cells. This study indicated the protection mechanism of Sirt1 in ischemic stress. Further research is still needed to elucidate the full picture of the role of Sirt1 in DNA repair under ischemic stroke conditions.

## Cerebral Blood Flow (CBF) Maintaining

Sirt1 can deacetylate endothelial nitric oxide synthase (eNOS) and then maintain CBF (Hattori et al., 2014). In one particular study, mice overexpressing Sirt1 were found to provide resistance to global ischemia by retaining cerebral perfusion up to 45%–50% of baseline data (Hattori et al., 2015). Moreover, Hattori et al. (2014) found that endothelial Sirt1 deacetylates and activates eNOS, thus normalizing CBF. They further suggested that Sirt1-eNOS-NO system is responsible for the suppression of Sirt1-induced cerebral hypoperfusion. Sirt1 overexpression significantly attenuated blood–brain barrier (BBB) disruption, and an interaction of Sirt1 with eNOS facilitated NO-dependent vascular relaxation (Hattori et al., 2014).

## Function on Energy Metabolism

The brain consumes more energy per gram of tissue than any other organ (Taxin et al., 2014). The state of ischemia in the brain confers onto energy exhaustion. In turn, this metabolic stress aggravates the ischemic injury. Therefore, to promote cell survival and improve ischemic injury in the brain, it is important to find defense mechanisms in energy exhaustion (Guo et al., 2017). AMP-activated protein kinase (AMPK) is considered to be a major metabolic energy sensor. Decreased cellular energy charge (increased AMP/ATP ratio) activates AMPK and then regulates energy metabolic homeostasis (Briski et al., 2014; Zainal Abidin et al., 2015). Pharmacologic inhibition of AMPK was reported to alleviate ischemic damage in an ischemia model (Li et al., 2007). Sirt1 is suggested to be associated with the regulation of energy metabolism (Boutant and Canto, 2014). Sirt1-related AMPK pathway was reported to protect against ischemia stroke in some studies (Wang et al., 2011).

Estrogen was proved to have beneficial effects under cerebral ischemic stress through Sirt1-AMPK signaling pathway. Estrogen deficiency is considered as a risk factor for ischemic stroke in females after menopause (Ahnstedt et al., 2016), which partly explains the stroke-related gender differences (Persky et al., 2010; Sohrabji et al., 2013). Guo et al. (2017) showed that upregulating Sirt1 expression and then promoting AMPK activation can further regulate energy exhaustion, finally contributing to neuron survival under ischemic stress. In addition to estrogen, the adipokynin hormone, leptin, plays a role in severe energy depletion. Avraham et al. (2010) found that the expression of Sirt1 gene increased in the cortex after leptin administration, which was in line with the reduction of infarct volume. This finding suggested that Sirt1 expression protects cortical neurons by modulating energetic status (Avraham et al., 2010).

Nicotinamide phosphoribosyltransferase (Nampt, also known as visfatin), the rate-limiting enzyme in mammalian NAD^+^ biosynthesis (Wang et al., 2012), plays several roles in protecting against ischemia, one of which was associated with the Nampt-Sirt1-AMPK neuroprotective signaling pathway. Wang et al. (2012) reported that Nampt was significantly upregulated in the penumbra and infarct core of MCAO models. Inhibition and overexpression of Nampt augmented and reduced the infarction in MCAO rats, respectively. The upregulation of Nampt positively modulated NAD^+^ levels and then upregulated Sirt1, contributing to LKB1 deacetylation, and thereby activating AMPK. This finding indicated that Nampt is an important protective factor in ischemic stroke (Wang et al., 2012).

Emerging studies showed that resveratrol could activate AMPK (Dasgupta and Milbrandt, 2007). Resveratrol was reported to activate a PDE-mediated signaling pathway that activates p-AMPK and Sirt1, thereby conferring cerebral ischemic tolerance (Wan et al., 2016).

The brain relies heavily on glucose for energy production (Koronowski et al., 2017). Production of ATP from glycolysis is crucial for fast axonal transport of vesicles (Zala et al., 2013), the energetic demand of action potential firing (Ashrafi et al., 2017), and the maintenance of synaptic ATP levels under energetic stress (Jang et al., 2016). Under ischemic conditions, it is important to utilize glucose more efficiently. Glycolysis can produce a significant amount of ATP to maintain ion gradients and delay depolarization. This effect is associated with neuronal Sirt1. One particular study demonstrated that resveratrol preconditioning increased glycolytic rate in a Sirt1-dependent manner in neurons, thereby combating energetic stress in ischemic conditions (Koronowski et al., 2017).

## Autophagy

Autophagic processes have been implicated as cell death mechanisms in the degradation and recycling of subcellular organelles (Kundu and Thompson, 2008). Generally, in the neuronal system, moderate autophagy is neuroprotective while inadequate or excess autophagy may lead to neuronal death (Shacka et al., 2008; Yue et al., 2008). Recently, autophagy has been recognized as a key process in ischemic stroke in addition to neurodegenerative diseases such as Alzheimer’s and Parkinson’s disease (Yue et al., 2008; Puyal et al., 2009). Sirt1 was first reported to regulate autophagy in Lee et al. (2008).

In addition to the Nampt-Sirt1-AMPK signaling pathway mentioned above, Nampt was demonstrated to have regulatory effects on autophagy under cerebral ischemic conditions (Wang et al., 2012). Wang et al. (2012) reported that Nampt promotes neuronal survival through inducing autophagy via regulating the TSC2-mTOR-S6K1 signaling pathway in a Sirt1-dependent manner during cerebral ischemia. Further studies are needed to find out more about the relationship between autophagy and ischemic stroke.

## Impact on Blood–Brain Barrier (BBB)

Although most studies suggest that Sirt1 is a protective mediator in ischemic stroke, there are still a few studies that contradict these findings. Chen et al. (2018) reported that the activation of Sirt1 was associated with increased BBB permeability through AMPK-PGC1. Disruption of BBB and the cerebral edema that follows are the key pathogenic events contributing to neurological dysfunction and cerebral infarct after ischemic stroke (Chen et al., 2018). It is important to investigate further to find out the detailed mechanism and explain the contradiction between such studies.

## Other Mechanisms

Some studies reported that Sirt1 is important in the protection against ischemic stroke. Caloric restriction (CR) is defined as approximately 30% reduction in caloric intake, without compromising the maintenance of all essential nutrients (Redman et al., 2007). Short-term food restriction (40% less food over a 3-month period) was reported to attenuate ischemia-induced damage and improve functional recovery following global ischemia (Roberge et al., 2008). Proper period of CR can protect neurons from focal ischemic injury (Duan et al., 2001). Caloric restriction was found to increase the synthesis of Sirt1 and reduce the downregulation of Sirt1 expression in MCAO (Ran et al., 2015). Another study found that CDP-choline (citicoline), an intermediate in the biosynthesis of phosphatidylcholine, acted as Sirt1 activator by upregulating its expression and thereby reducing infarct volume in ischemic models (Hurtado et al., 2013). Further studies are still needed to elucidate the specific mechanisms.

## Conclusion and Prospects for Future Research

Since Sirt1 could exacerbate energy depletion and is associated with increased BBB permeability, there is still a controversy whether choosing Sirt1 as a treatment target and increasing the Sirt1 level would benefit the cerebral tissue under ischemic stress. Therefore, additional studies investigating the role of Sirt1 in ischemic stroke involving different cerebral cell types and animal models are necessary to arrive at more convincing conclusions. Besides, the relationship between Sirt1 genetic polymorphism and ischemic stroke has not been researched extensively, which also needs further exploration. Altogether, Sirt1 is a promising therapeutic target for ischemic stroke for attenuating ischemic stress and improving stroke outcome.

## Author Contributions

All authors listed have made a substantial, direct and intellectual contribution to the work, and approved it for publication.

## Conflict of Interest Statement

The authors declare that the research was conducted in the absence of any commercial or financial relationships that could be construed as a potential conflict of interest.
